# Crystal structure of 3-(prop-2-en-1-yl)-1-{[(1*E*)-1,2,3,4-tetra­hydro­naphthalen-1-yl­idene]amino}­thio­urea

**DOI:** 10.1107/S2056989015021076

**Published:** 2015-11-21

**Authors:** Joel T. Mague, Shaaban K. Mohamed, Mehmet Akkurt, Alaa A Hassan, Ahmed T. Abdel-Aziz, Mustafa R. Albayati

**Affiliations:** a Department of Chemistry, Tulane University, New Orleans, LA 70118, USA; b Faculty of Science & Engineering, School of Healthcare Science, Manchester Metropolitan University, Manchester M1 5GD, England; c Chemistry Department, Faculty of Science, Minia University, 61519 El-Minia, Egypt; d Department of Physics, Faculty of Sciences, Erciyes University, 38039 Kayseri, Turkey; e Kirkuk University, College of Education, Department of Chemistry, Kirkuk, Iraq

**Keywords:** thio­semicarbazone, crystal structure, N—H⋯S hydrogen bond

## Abstract

In the title compound, C_14_H_17_N_3_S, the dihedral angle between the planes of the benzene ring and the thio­semicarbazone group (r.m.s. deviation = 0.031 Å) is 8.45 (4)°. A short intra­molcular N—H⋯N contact is seen. In the crystal, weak N—H⋯S hydrogen bonds connect the mol­ecules into *C*(4) chains propagating in the [010] direction, with adjacent mol­ecules in the chain related by 2_1_ screw-axis symmetry.

## Related literature   

For a related structure and background to thio­semi­car­ba­zones, see: Mohamed *et al.* (2015 ▸). For further synthetic details, see: Mague *et al.* (2014 ▸).
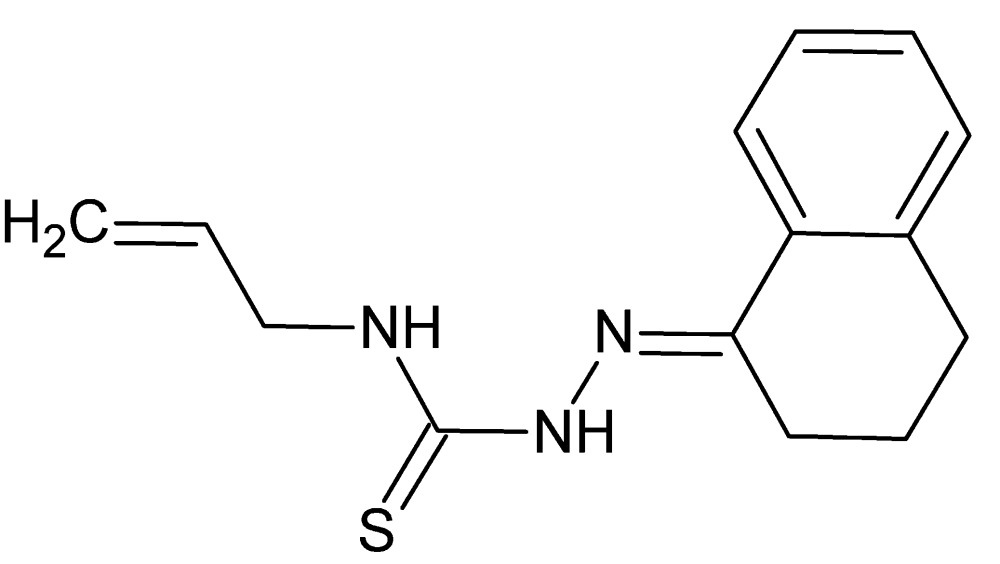


## Experimental   

### Crystal data  

C_14_H_17_N_3_S
*M* *_r_* = 259.36
Monoclinic, 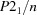

*a* = 7.6665 (2) Å
*b* = 8.5788 (2) Å
*c* = 20.4072 (5) Å
β = 91.794 (1)°
*V* = 1341.51 (6) Å^3^
*Z* = 4
Cu *K*α radiation
μ = 2.02 mm^−1^
*T* = 150 K
0.20 × 0.19 × 0.16 mm

### Data collection  

Bruker D8 VENTURE PHOTON 100 CMOS diffractometer
Absorption correction: multi-scan (*SADABS*; Bruker, 2014 ▸) *T*_min_ = 0.68, *T*_max_ = 0.73
10141 measured reflections
2687 independent reflections
2486 reflections with *I* > 2σ(*I*)
*R*_int_ = 0.022

### Refinement  

*R*[*F* ^2^ > 2σ(*F* ^2^)] = 0.031
*wR*(*F* ^2^) = 0.084
*S* = 1.07
2687 reflections
163 parameters
H-atom parameters constrained
Δρ_max_ = 0.25 e Å^−3^
Δρ_min_ = −0.24 e Å^−3^

### 

Data collection: *APEX2* (Bruker, 2014 ▸); cell refinement: *SAINT* (Bruker, 2014 ▸); data reduction: *SAINT*; program(s) used to solve structure: *SHELXT* (Bruker, 2014 ▸); program(s) used to refine structure: *SHELXL2014* (Sheldrick, 2015 ▸); molecular graphics: *DIAMOND* (Brandenburg & Putz, 2012 ▸); software used to prepare material for publication: *SHELXTL* (Bruker, 2014 ▸).

## Supplementary Material

Crystal structure: contains datablock(s) global, I. DOI: 10.1107/S2056989015021076/hb7539sup1.cif

Structure factors: contains datablock(s) I. DOI: 10.1107/S2056989015021076/hb7539Isup2.hkl

Supporting information file. DOI: 10.1107/S2056989015021076/hb7539Isup3.cml

**Fig. 1.**
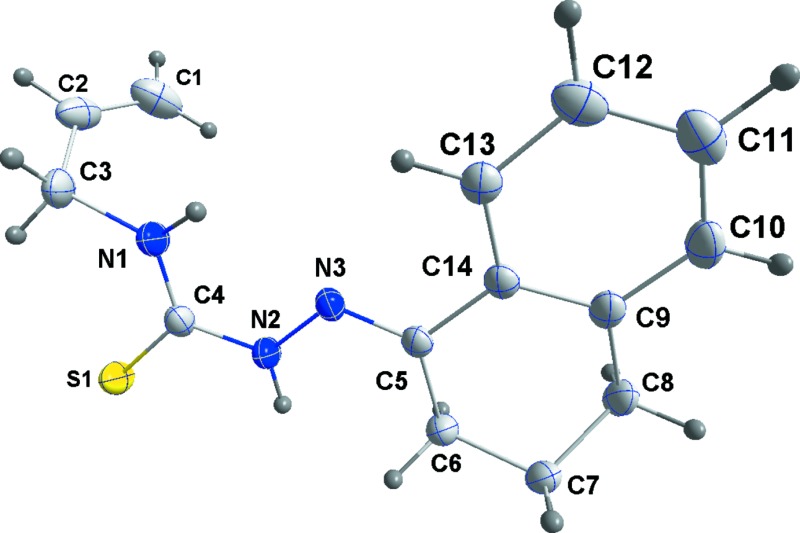
The title molecule with 50% probability displacement ellipsoids.

**Fig. 2.**
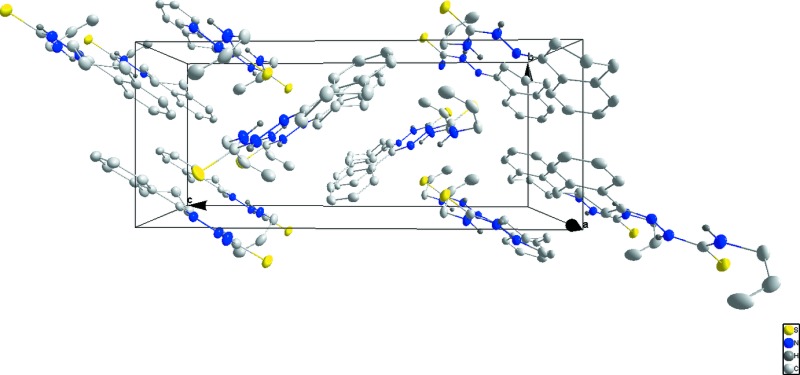
Packing viewed down the a axis.

. DOI: 10.1107/S2056989015021076/hb7539fig1.tif
The title mol­ecule with 50% probability displacement ellipsoids.

a . DOI: 10.1107/S2056989015021076/hb7539fig2.tif
Packing viewed down the *a* axis.

CCDC reference: 1435398

Additional supporting information:  crystallographic information; 3D view; checkCIF report

## Figures and Tables

**Table 1:** Hydrogen-bond geometry (Å, °)

*D*—H⋯*A*	*D*—H	H⋯*A*	*D*⋯*A*	*D*—H⋯*A*
N1—H1N⋯N3	0.91	2.17	2.6146 (13)	109
N1—H1N⋯S1^i^	0.91	2.82	3.4642 (11)	129

Symmetry code: (i) 
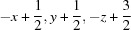
.

## supplementary crystallographic information

### S1. Experimental

The title compound was prepared according to our recently reported method (Mague *et al.*, 2014). Colourless blocks were recrystallised from ethanol solution. M.p. 393–394 K, 92% yield.

### S2. Refinement

H-atoms attached to carbon were placed in calculated positions (C—H = 0.95 - 0.99 Å) while those attached to nitrogen were placed in locations derived from a difference map and their parameters adjusted to give N—H = 0.91 Å. All were included as riding contributions with isotropic displacement parameters 1.2 times those of the attached atoms.

### Crystal data

**Table:**

C_14_H_17_N_3_S	*F*(000) = 552
*M**_r_* = 259.36	*D*_x_ = 1.284 Mg m^−^^3^
Monoclinic, *P*2_1_/*n*	Cu *K*α radiation, λ = 1.54178 Å
*a* = 7.6665 (2) Å	Cell parameters from 8207 reflections
*b* = 8.5788 (2) Å	θ = 4.3–74.5°
*c* = 20.4072 (5) Å	µ = 2.02 mm^−^^1^
β = 91.794 (1)°	*T* = 150 K
*V* = 1341.51 (6) Å^3^	Block, colourless
*Z* = 4	0.20 × 0.19 × 0.16 mm

### Data collection

**Table:**

Bruker D8 VENTURE PHOTON 100 CMOS diffractometer	2687 independent reflections
Radiation source: INCOATEC IµS micro–focus source	2486 reflections with *I* > 2σ(*I*)
Mirror monochromator	*R*_int_ = 0.022
Detector resolution: 10.4167 pixels mm^-1^	θ_max_ = 74.5°, θ_min_ = 4.3°
ω scans	*h* = −8→9
Absorption correction: multi-scan (*SADABS*; Bruker, 2014)	*k* = −10→10
*T*_min_ = 0.68, *T*_max_ = 0.73	*l* = −21→25
10141 measured reflections	

### Refinement

**Table:**

Refinement on *F*^2^	Primary atom site location: structure-invariant direct methods
Least-squares matrix: full	Secondary atom site location: difference Fourier map
*R*[*F*^2^ > 2σ(*F*^2^)] = 0.031	Hydrogen site location: mixed
*wR*(*F*^2^) = 0.084	H-atom parameters constrained
*S* = 1.07	*w* = 1/[σ^2^(*F*_o_^2^) + (0.0458*P*)^2^ + 0.3454*P*] where *P* = (*F*_o_^2^ + 2*F*_c_^2^)/3
2687 reflections	(Δ/σ)_max_ = 0.001
163 parameters	Δρ_max_ = 0.25 e Å^−^^3^
0 restraints	Δρ_min_ = −0.24 e Å^−^^3^

### Special details

**Table:**

Geometry. All e.s.d.'s (except the e.s.d. in the dihedral angle between two l.s. planes) are estimated using the full covariance matrix. The cell e.s.d.'s are taken into account individually in the estimation of e.s.d.'s in distances, angles and torsion angles; correlations between e.s.d.'s in cell parameters are only used when they are defined by crystal symmetry. An approximate (isotropic) treatment of cell e.s.d.'s is used for estimating e.s.d.'s involving l.s. planes.
Refinement. Refinement of *F*^2^ against ALL reflections. The weighted *R*-factor *wR* and goodness of fit *S* are based on *F*^2^, conventional *R*-factors *R* are based on *F*, with *F* set to zero for negative *F*^2^. The threshold expression of *F*^2^ > σ(*F*^2^) is used only for calculating *R*-factors(gt) *etc*. and is not relevant to the choice of reflections for refinement. *R*-factors based on *F*^2^ are statistically about twice as large as those based on *F*, and *R*- factors based on ALL data will be even larger. H-atoms attached to carbon were placed in calculated positions (C—H = 0.95 - 0.99 Å) while those attached to nitrogen were placed in locations derived from a difference map and their parameters adjusted to give N—H = 0.91 Å. All were included as riding contributions with isotropic displacement parameters 1.2 times those of the attached atoms.

### Fractional atomic coordinates and isotropic or equivalent isotropic displacement parameters (Å^2^)

**Table:**

	*x*	*y*	*z*	*U*_iso_*/*U*_eq_	
S1	0.48923 (4)	0.32351 (4)	0.80129 (2)	0.03037 (11)	
N1	0.24491 (13)	0.48661 (13)	0.73353 (5)	0.0280 (2)	
H1N	0.2236	0.5554	0.7003	0.034*	
N2	0.51325 (12)	0.47130 (12)	0.68903 (5)	0.0239 (2)	
H2N	0.6272	0.4415	0.6911	0.029*	
N3	0.44987 (12)	0.56539 (11)	0.63901 (4)	0.0220 (2)	
C1	−0.0358 (2)	0.27861 (18)	0.69916 (8)	0.0472 (4)	
H1A	0.0572	0.2901	0.6699	0.057*	
H1B	−0.1320	0.2131	0.6878	0.057*	
C2	−0.03150 (17)	0.35274 (17)	0.75504 (7)	0.0348 (3)	
H2	−0.1277	0.3373	0.7826	0.042*	
C3	0.10929 (16)	0.45938 (16)	0.78005 (6)	0.0299 (3)	
H3A	0.0565	0.5606	0.7917	0.036*	
H3B	0.1628	0.4143	0.8206	0.036*	
C4	0.40678 (15)	0.43269 (13)	0.73855 (5)	0.0234 (2)	
C5	0.55646 (14)	0.60710 (13)	0.59461 (5)	0.0203 (2)	
C6	0.74503 (15)	0.55855 (14)	0.59361 (6)	0.0249 (2)	
H6A	0.7515	0.4483	0.5795	0.030*	
H6B	0.7964	0.5655	0.6386	0.030*	
C7	0.85274 (15)	0.65815 (15)	0.54803 (6)	0.0265 (3)	
H7A	0.8674	0.7639	0.5669	0.032*	
H7B	0.9700	0.6114	0.5440	0.032*	
C8	0.76392 (16)	0.66981 (15)	0.48046 (6)	0.0279 (3)	
H8A	0.8331	0.7386	0.4522	0.033*	
H8B	0.7588	0.5652	0.4600	0.033*	
C9	0.58186 (15)	0.73399 (13)	0.48518 (5)	0.0235 (2)	
C10	0.50909 (18)	0.82536 (14)	0.43472 (6)	0.0293 (3)	
H10	0.5748	0.8447	0.3969	0.035*	
C11	0.34410 (18)	0.88813 (14)	0.43851 (6)	0.0323 (3)	
H11	0.2971	0.9499	0.4036	0.039*	
C12	0.24694 (17)	0.86062 (14)	0.49371 (6)	0.0299 (3)	
H12	0.1333	0.9037	0.4966	0.036*	
C13	0.31603 (15)	0.77037 (13)	0.54449 (6)	0.0244 (2)	
H13	0.2494	0.7522	0.5822	0.029*	
C14	0.48339 (15)	0.70551 (13)	0.54080 (5)	0.0208 (2)	

### Atomic displacement parameters (Å^2^)

**Table:**

	*U*^11^	*U*^22^	*U*^33^	*U*^12^	*U*^13^	*U*^23^
S1	0.02807 (18)	0.03706 (19)	0.02591 (18)	−0.00248 (11)	−0.00050 (12)	0.01231 (11)
N1	0.0248 (5)	0.0385 (6)	0.0208 (5)	−0.0010 (4)	0.0016 (4)	0.0074 (4)
N2	0.0233 (5)	0.0280 (5)	0.0204 (5)	−0.0003 (4)	0.0011 (4)	0.0048 (4)
N3	0.0247 (5)	0.0240 (5)	0.0173 (4)	−0.0012 (4)	−0.0001 (4)	0.0013 (3)
C1	0.0498 (9)	0.0378 (8)	0.0528 (9)	−0.0060 (7)	−0.0182 (7)	0.0063 (7)
C2	0.0252 (6)	0.0413 (7)	0.0378 (7)	−0.0005 (5)	−0.0005 (5)	0.0176 (6)
C3	0.0288 (6)	0.0410 (7)	0.0203 (6)	0.0011 (5)	0.0056 (5)	0.0035 (5)
C4	0.0259 (6)	0.0247 (6)	0.0196 (5)	−0.0047 (4)	0.0001 (4)	−0.0001 (4)
C5	0.0228 (5)	0.0206 (5)	0.0176 (5)	−0.0034 (4)	−0.0004 (4)	−0.0024 (4)
C6	0.0223 (5)	0.0273 (6)	0.0249 (6)	−0.0018 (4)	0.0000 (4)	0.0021 (4)
C7	0.0216 (6)	0.0329 (6)	0.0250 (6)	−0.0057 (5)	0.0012 (5)	−0.0015 (5)
C8	0.0267 (6)	0.0362 (7)	0.0209 (6)	−0.0066 (5)	0.0043 (5)	−0.0025 (4)
C9	0.0278 (6)	0.0230 (5)	0.0198 (5)	−0.0077 (4)	0.0001 (4)	−0.0018 (4)
C10	0.0389 (7)	0.0298 (6)	0.0192 (6)	−0.0094 (5)	−0.0003 (5)	0.0019 (4)
C11	0.0450 (7)	0.0239 (6)	0.0272 (6)	−0.0038 (5)	−0.0098 (5)	0.0041 (5)
C12	0.0315 (6)	0.0237 (6)	0.0339 (7)	0.0025 (5)	−0.0068 (5)	−0.0013 (5)
C13	0.0263 (6)	0.0233 (5)	0.0235 (6)	−0.0024 (4)	0.0005 (4)	−0.0016 (4)
C14	0.0238 (5)	0.0198 (5)	0.0186 (5)	−0.0039 (4)	−0.0014 (4)	−0.0018 (4)

### Geometric parameters (Å, º)

**Table:**

S1—C4	1.6928 (12)	C6—H6A	0.9900
N1—C4	1.3254 (16)	C6—H6B	0.9900
N1—C3	1.4487 (16)	C7—C8	1.5220 (16)
N1—H1N	0.9098	C7—H7A	0.9900
N2—C4	1.3597 (15)	C7—H7B	0.9900
N2—N3	1.3778 (13)	C8—C9	1.5063 (17)
N2—H2N	0.9098	C8—H8A	0.9900
N3—C5	1.2897 (15)	C8—H8B	0.9900
C1—C2	1.305 (2)	C9—C10	1.3964 (17)
C1—H1A	0.9500	C9—C14	1.4042 (16)
C1—H1B	0.9500	C10—C11	1.379 (2)
C2—C3	1.4926 (19)	C10—H10	0.9500
C2—H2	0.9500	C11—C12	1.390 (2)
C3—H3A	0.9900	C11—H11	0.9500
C3—H3B	0.9900	C12—C13	1.3854 (17)
C5—C14	1.4811 (15)	C12—H12	0.9500
C5—C6	1.5052 (16)	C13—C14	1.4027 (16)
C6—C7	1.5249 (16)	C13—H13	0.9500
			
C4—N1—C3	125.63 (10)	C8—C7—C6	110.73 (10)
C4—N1—H1N	115.4	C8—C7—H7A	109.5
C3—N1—H1N	118.5	C6—C7—H7A	109.5
C4—N2—N3	119.17 (10)	C8—C7—H7B	109.5
C4—N2—H2N	119.7	C6—C7—H7B	109.5
N3—N2—H2N	121.0	H7A—C7—H7B	108.1
C5—N3—N2	117.81 (10)	C9—C8—C7	110.81 (10)
C2—C1—H1A	120.0	C9—C8—H8A	109.5
C2—C1—H1B	120.0	C7—C8—H8A	109.5
H1A—C1—H1B	120.0	C9—C8—H8B	109.5
C1—C2—C3	126.56 (14)	C7—C8—H8B	109.5
C1—C2—H2	116.7	H8A—C8—H8B	108.1
C3—C2—H2	116.7	C10—C9—C14	118.77 (11)
N1—C3—C2	113.63 (11)	C10—C9—C8	120.51 (11)
N1—C3—H3A	108.8	C14—C9—C8	120.71 (10)
C2—C3—H3A	108.8	C11—C10—C9	121.58 (12)
N1—C3—H3B	108.8	C11—C10—H10	119.2
C2—C3—H3B	108.8	C9—C10—H10	119.2
H3A—C3—H3B	107.7	C10—C11—C12	119.66 (11)
N1—C4—N2	116.10 (10)	C10—C11—H11	120.2
N1—C4—S1	125.31 (9)	C12—C11—H11	120.2
N2—C4—S1	118.58 (9)	C13—C12—C11	119.94 (12)
N3—C5—C14	116.47 (10)	C13—C12—H12	120.0
N3—C5—C6	124.24 (10)	C11—C12—H12	120.0
C14—C5—C6	119.26 (10)	C12—C13—C14	120.69 (11)
C5—C6—C7	113.07 (10)	C12—C13—H13	119.7
C5—C6—H6A	109.0	C14—C13—H13	119.7
C7—C6—H6A	109.0	C13—C14—C9	119.35 (10)
C5—C6—H6B	109.0	C13—C14—C5	120.78 (10)
C7—C6—H6B	109.0	C9—C14—C5	119.87 (10)
H6A—C6—H6B	107.8		
			
C4—N2—N3—C5	176.28 (10)	C14—C9—C10—C11	0.25 (17)
C4—N1—C3—C2	−109.16 (14)	C8—C9—C10—C11	−178.62 (11)
C1—C2—C3—N1	4.4 (2)	C9—C10—C11—C12	0.07 (18)
C3—N1—C4—N2	179.56 (11)	C10—C11—C12—C13	−0.07 (18)
C3—N1—C4—S1	−1.23 (18)	C11—C12—C13—C14	−0.26 (18)
N3—N2—C4—N1	1.89 (16)	C12—C13—C14—C9	0.58 (17)
N3—N2—C4—S1	−177.38 (8)	C12—C13—C14—C5	−179.10 (10)
N2—N3—C5—C14	178.78 (9)	C10—C9—C14—C13	−0.57 (16)
N2—N3—C5—C6	0.54 (16)	C8—C9—C14—C13	178.30 (10)
N3—C5—C6—C7	−163.89 (11)	C10—C9—C14—C5	179.12 (10)
C14—C5—C6—C7	17.91 (14)	C8—C9—C14—C5	−2.02 (16)
C5—C6—C7—C8	−50.50 (14)	N3—C5—C14—C13	10.37 (15)
C6—C7—C8—C9	56.66 (13)	C6—C5—C14—C13	−171.29 (10)
C7—C8—C9—C10	147.83 (11)	N3—C5—C14—C9	−169.31 (10)
C7—C8—C9—C14	−31.02 (15)	C6—C5—C14—C9	9.03 (15)

### Hydrogen-bond geometry (Å, º)

**Table:**

*D*—H···*A*	*D*—H	H···*A*	*D*···*A*	*D*—H···*A*
N1—H1*N*···N3	0.91	2.17	2.6146 (13)	109
N1—H1*N*···S1^i^	0.91	2.82	3.4642 (11)	129

Symmetry code: (i) −*x*+1/2, *y*+1/2, −*z*+3/2.
