# Supplementary figures and images for: Crystal structure of 3-(prop-2-en-1-yl)-1-{[(1E)-1,2,3,4-tetra­hydro­naphthalen-1-yl­idene]amino}­thio­urea
Source: Acta Crystallogr E Crystallogr Commun. 2015 Nov 21;71(Pt 12):o976–7. doi: 10.1107/S2056989015021076 (PMC4719931; doi:10.1107/S2056989015021076)

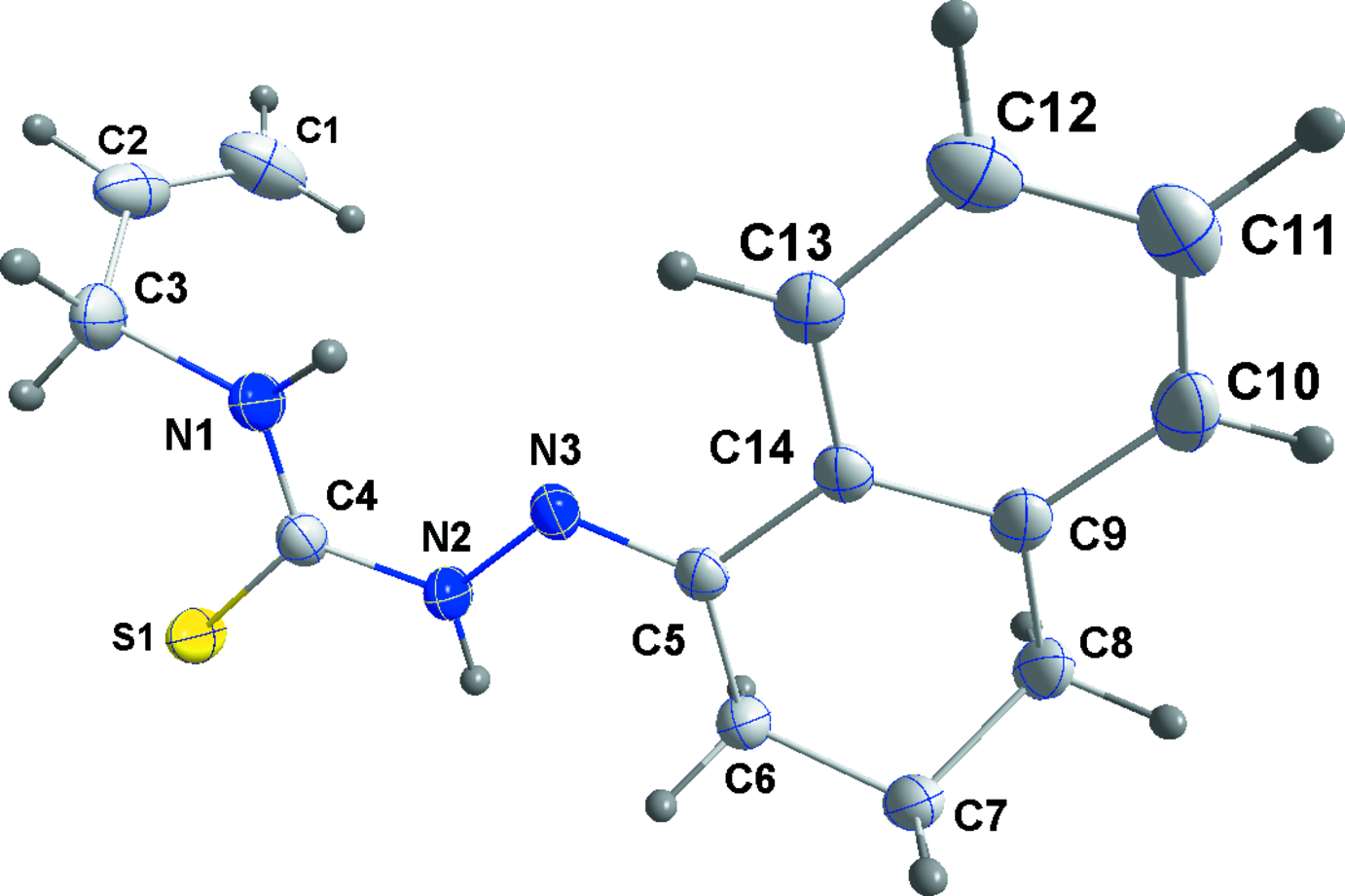

Supplement: Supplementary file 4 [file e-71-0o976-fig1.tif]

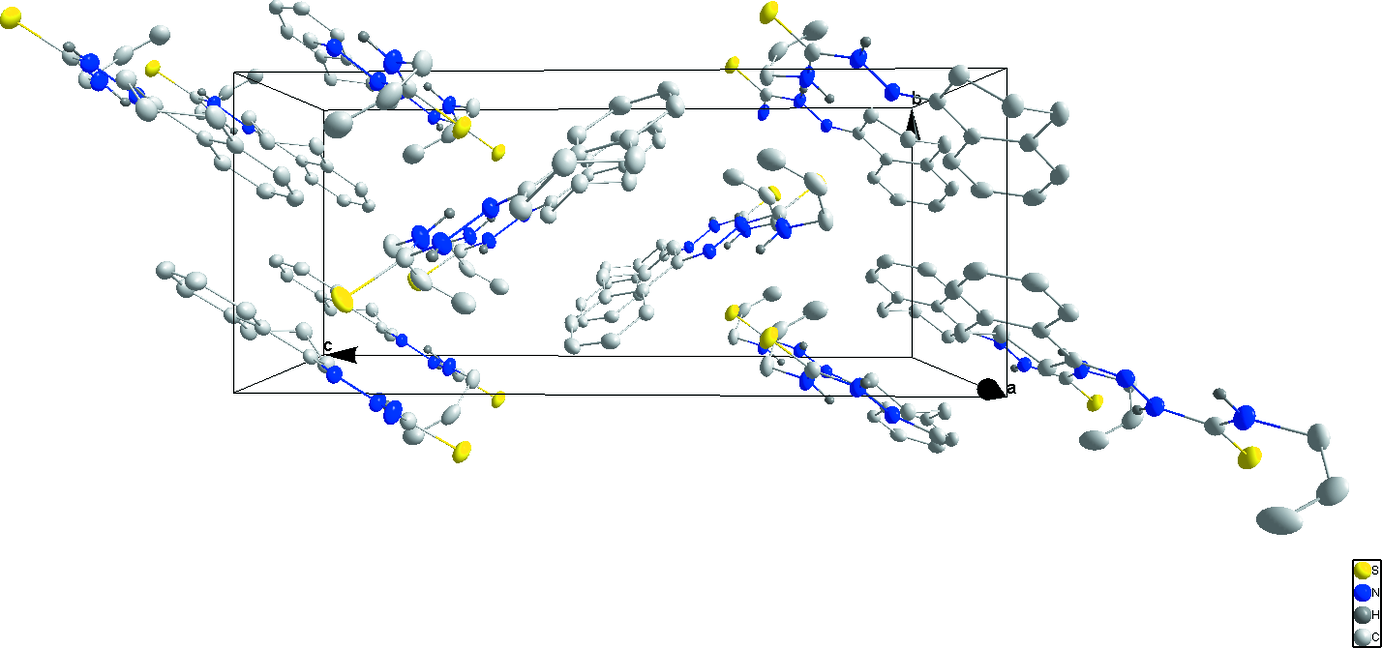

Supplement: Supplementary file 5 [file e-71-0o976-fig2.tif]
